# Complete mitogenome of the entomopathogenic fungus *Akanthomyces lecanii*

**DOI:** 10.1080/23802359.2020.1721349

**Published:** 2020-02-03

**Authors:** Yong-Jie Zhang, Xue-Bin Yang, Shu Zhang

**Affiliations:** a School of Life Science, Shanxi University, Taiyuan, China;; b Institute of Applied Chemistry, Shanxi University, Taiyuan, China

**Keywords:** *Akanthomyces lecanii*, *Cordyceps confragosa*, *Lecanicillium lecanii*, mitogenome, Cordycipitaceae

## Abstract

In this study, the complete mitogenome of an entomopathogenic fungus *Akanthomyces lecanii* is assembled and annotated. This circular mitogenome is 24,643 bp in length and consists of 2 rRNA genes (*rnl* and *rns*), 26 tRNA genes and 14 standard protein-coding genes of the oxidative phosphorylation system. Only one intron (group IA) is identified, which invades *rnl* and carries an ORF coding for ribosomal protein S3. Phylogenetic analysis based on concatenated mitochondrial nucleotide sequences confirms *A. lecanii* in Cordycipitaceae, and *A. lecanii* clusters together with *Akanthomyces muscarius*.

*Akanthomyces lecanii* (Zimm.) Spatafora, Kepler & B. Shrestha is a fungus affiliated to Cordycipitaceae, Hypocreales, Sordariomycetes, Ascomycota. It is previously known as *Cordyceps confragosa*, *Torrubiella confragosa*, *Lecanicillium lecanii*, etc (Kepler et al. 2017). This species is of interest because it is capable of infecting various pest insects (esp. scale insects), has a broad geographical distribution, and shows promise in commercial development (Goettel et al. 2008). This fungus also shows potential against plant-parasitic nematodes and plant fungal diseases (e.g., strawberry powdery mildew, coffee leaf rust) (Miller et al. 2004; Shinya et al. 2008; Vandermeer et al. 2009). The nuclear genome of *A. lecanii* has been reported using the strain RCEF 1005 (Shang et al. 2016). Nevertheless, the mitogenome information of the fungus is still lacking. Herein, we present the complete mitogenome of *A. lecanii* RCEF 1005. This strain was isolated from a lepidopteran larva in Yuexi, Anhui, China (N30°51′, E116°21′) and was deposited in the culture collection of the Research Center on Entomogenous Fungi (RCEF), Anhui Agricultural University, Hefei, China.

Total DNA of RCEF 1005 was fragmented by sonication to a size of ∼280 bp, followed by sequencing on an Illumina NovaSeq platform in 2 × 150 bp reads. Mitogenome was *de novo* assembled from clean reads using NOVOPlasty (Dierckxsens et al. 2017) and then annotated as described previously (Zhang et al. 2017).

The mitogenome of *A. lecanii* (GenBank accession: MN904747) is a circular molecule of 24,643 bp with an AT content of 72.8%. It is rather compact with genic regions (21,232 bp) accounting for 86.2% of the total bases. This mitogenome encodes two ribosomal RNAs (*rnl* and *rns*), 26 tRNAs, 14 conserved proteins of the oxidative phosphorylation system (*nad1*-*6*, *4 L*; *cob*; *cox1*-*3*, and *atp6*, *8*, *9*). These tRNA genes code for all 20 standard amino acids. Among them, there are three tRNA genes for methionine with the same anticodon, two tRNA genes for arginine, isoleucine, leucine, and serine with different anticodons. The majority of tRNA genes are clustered upstream (*trnV*, *I2*, *S2*, *W*, *P*) and downstream (*trnT*, *E*, *M1*, *M2*, *L1*, *A*, *F*, *K*, *L2*, *Q*, *H*, *M3*) of the *rnl* gene, and downstream (*trnY*, *D*, *S1*, *N, I1*) of the *rns* gene.

For the two neighboring gene pairs, *nad2/nad3* and *nad4L/nad5*, corresponding genes overlap by one nucleotide, which is characteristic of Cordycipitaceae (Fan et al. 2019). Not an intergenic free-standing ORF is identified. Only one intron is present in the mitogenome. This group IA intron (designated as mL2450) interrupts *rnl* and contains an ORF (*orf447*) encoding ribosomal protein S3.

Phylogenetic analysis based on mitochondrial nucleotide sequences confirms *A. lecanii* as a member of Cordycipitaceae (Figure 1). The fungus is closely related to *Akanthomyces muscarius*, and their clustering received 100% support. This result supports the recent renaming of *Cordyceps confragosa* to *Akanthomyces lecanii* (Kepler et al. 2017). It should be noted that *Cordyceps confragosa* is still the name currently accepted by the NCBI taxonomy (https://www.ncbi.nlm.nih.gov/taxonomy/). A recent molecular phylogenetic investigation proposed 11 names that should be maintained and 8 names that should be rejected in Cordycipitaceae (Kepler et al. 2017). To date, only four of these recommended genera (i.e., *Akanthomyces*, *Beauveria*, *Cordyceps* and *Parengyodontium*) have representative species with available mitogenomes. Mitogenomes from species in other proposed genera need to be sequenced. Our phylogeny also supports the generic name changes in Cordycipitaceae because all species in *Akanthomyces*, *Beauveria* and *Cordyceps* each formed an individual group (Figure 1). Two exceptions are *Isaria farinosa* ARSEF3 and *Lecanicillium saksenae*. These two species remain to be assessed and renamed in compliance with the new taxonomic revision since *Isaria* and *Lecanicillium* are among the suppressed generic names.

**Figure 1.**
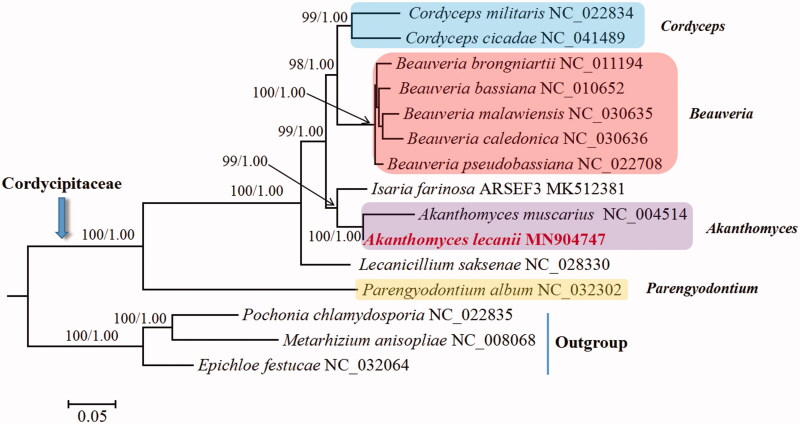
Phylogenetic analysis of Cordycipitaceae species based on 13 mitochondrial protein-encoding genes. Concatenated nucleotide sequences of *atp6*, *atp8*, *atp9*, *cob*, *cox1*, *cox2*, *cox3*, *nad1*, *nad2*, *nad3*, *nad4*, *nad4L* and *nad6*, a total of 10,503 characters, were used. The gene *nad5* was excluded due to its significant phylogenetic conflict with *cox1* and *nad3*. Three Clavicipitaceae species (*Epichloe festucae*, *Metarhizium anisopliae* and *Pochonia chlamydosporia*) were used as outgroups. The tree shown here was the single best topology recovered from the maximum likelihood (ML) approach as implemented in RAxML v8.2.12, and the topology was identical to that recovered from Bayesian inference (BI) as implemented in MrBayes v3.2.7. Support values from ML (*before forward slash*) and BI (*after forward slash*) analyses were given for nodes that received bootstrap values ≥70% (for ML) or posterior probability ≥0.95 (for BI).
